# Crystal structure of 2-(2,4-diphenyl-3-aza­bicyclo­[3.3.1]nonan-9-yl­idene)aceto­nitrile

**DOI:** 10.1107/S2056989015017740

**Published:** 2015-09-26

**Authors:** K. Priya, K. Saravanan, S. Kabilan, S. Selvanayagam

**Affiliations:** a Department of Chemistry, Annamalai University, Annamalainagar 608 002, India; b PG & Research Department of Physics, Government Arts College, Melur 625 106, India

**Keywords:** crystal structure, 3-aza­bicyclo­nonane derivatives, chair conformation

## Abstract

In the title 3-aza­bicyclo­nonane derivative, C_22_H_22_N_2_, both the fused piperidine and cyclo­hexane rings adopt a chair conformation. The phenyl rings attached to the central aza­bicylononane fragment in an equatorial orientation are inclined to each other at 23.7 (1)°. The amino group is not involved in any hydrogen bonding, so the crystal packing is stabilized only by van der Waals forces.

## Related literature   

For the biological activities of 3-aza­bicyclo­nonane derivatives, see: Silver *et al.* (1967 ▸); Fleming & Wang (2003 ▸); Miller & Manson (2001 ▸); Fatiadi (1983 ▸). For related structures, see: Parthiban *et al.* (2008*a* ▸,*b* ▸,*c* ▸,*d* ▸,*e* ▸).
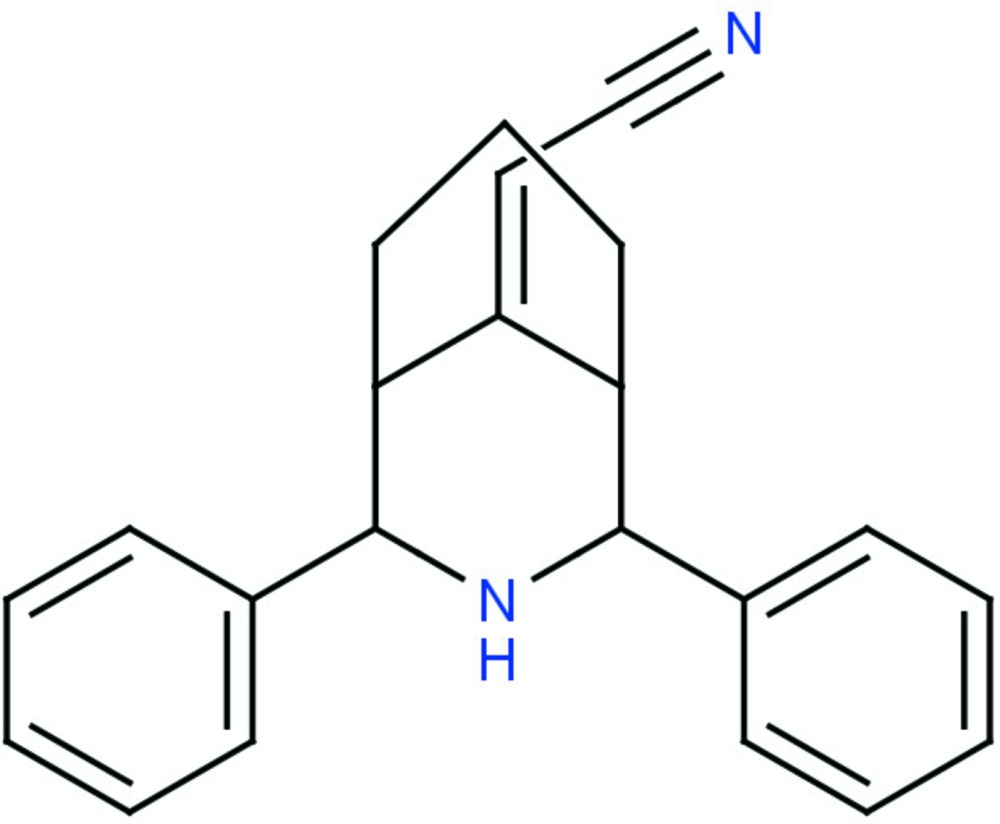


## Experimental   

### Crystal data  

C_22_H_22_N_2_
*M* *_r_* = 314.41
Triclinic, 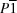

*a* = 7.9672 (5) Å
*b* = 8.3129 (5) Å
*c* = 13.6069 (8) Å
α = 89.607 (4)°
β = 81.886 (4)°
γ = 84.469 (4)°
*V* = 888.00 (9) Å^3^
*Z* = 2
Mo *K*α radiation
μ = 0.07 mm^−1^
*T* = 296 K
0.23 × 0.21 × 0.19 mm

### Data collection  

Bruker SMART APEX CCD area-detector diffractometer
14326 measured reflections
3814 independent reflections
2375 reflections with *I* > 2σ(*I*)
*R*_int_ = 0.034

### Refinement  

*R*[*F* ^2^ > 2σ(*F* ^2^)] = 0.047
*wR*(*F* ^2^) = 0.131
*S* = 1.05
3814 reflections
221 parameters
H atoms treated by a mixture of independent and constrained refinement
Δρ_max_ = 0.13 e Å^−3^
Δρ_min_ = −0.16 e Å^−3^

### 

Data collection: *SMART* (Bruker, 2001 ▸); cell refinement: *SAINT* (Bruker, 2001 ▸); data reduction: *SAINT*; program(s) used to solve structure: *SHELXS97* (Sheldrick, 2008 ▸); program(s) used to refine structure: *SHELXL2014*/7 (Sheldrick, 2015 ▸); molecular graphics: *ORTEP-3 for Windows* (Farrugia, 2012 ▸) and *PLATON* (Spek, 2009 ▸); software used to prepare material for publication: *SHELXL2014*/7 and *PLATON*.

## Supplementary Material

Crystal structure: contains datablock(s) I, global. DOI: 10.1107/S2056989015017740/cv5495sup1.cif

Structure factors: contains datablock(s) I. DOI: 10.1107/S2056989015017740/cv5495Isup2.hkl

Supporting information file. DOI: 10.1107/S2056989015017740/cv5495Isup3.cml

**Fig. 1.**
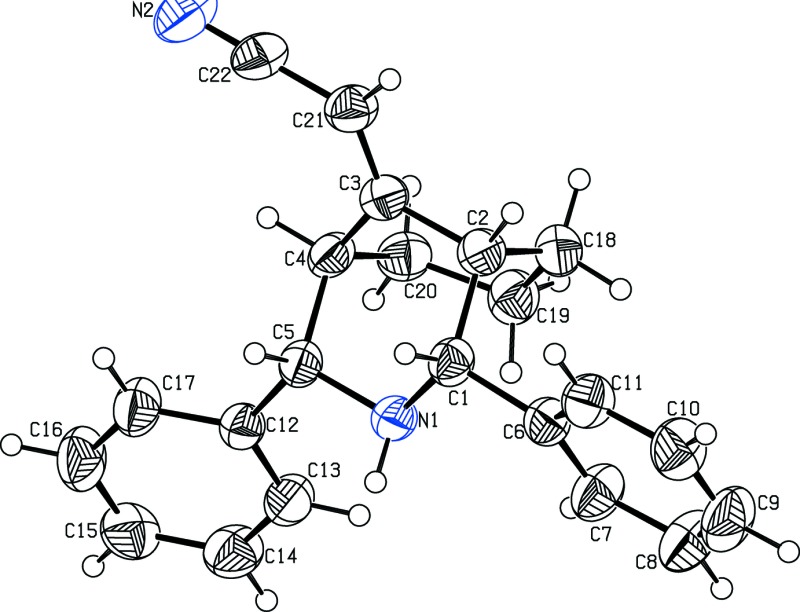
The molecular structure of the title compound with atom labelling. Displacement ellipsoids are drawn at the 40% probability level.

. DOI: 10.1107/S2056989015017740/cv5495fig1.tif
The mol­ecular structure of the title compound with atom labelling. Displacement ellipsoids are drawn at the 40% probability level.

CCDC reference: 1426330

Additional supporting information:  crystallographic information; 3D view; checkCIF report

## supplementary crystallographic information

### S1. Chemical context

Nitrile derivatives received considerable inter­est since they have been used in biological field as well as in optical fields (Silver *et al.*, 1967). Alkenyl nitriles are unique structural units and versatile building blocks in organic synthesis for natural products, pharmaceuticals, agricultural chemicals, and dyes (Fleming & Wang, 2003; Miller & Manson,2001; Fatiadi, 1983). Hence, the synthesis and stereochemistry of 3-aza­bicyclo­nonan-9-ones are under intensive study (Parthiban *et al.*, 2008a, b, c, d, e). In continuation of our work with 3-aza­bicyclo­nonane derivatives, we have undertaken the crystal structure determination of the title compound, and the results are presented here.

### S2. Structural commentary

The molecular structure of the title compound is shown in Fig. 1. The bond length C22—N2 of 1.146 (2) Å confirms the triple bond character. Two phenyl rings attached to the 3-aza­biclononane fragment form a dihedral angle of 23.7 (1)°. The piperidine (N1/C1—C5) and cyclo­hexane (C2—C4/C20/C19/C18) rings adopt chair conformation. This is confirmed by the puckering parameters q_2_ = 0.044 (1) Å, q_3_ = 0.598 (1) Å, Q_T_ = 0.600 (1) Å, φ = -156.7 (4)° for piperidine ring, and q_2_ = 0.122 (1) Å, q_3_ = -0.556 (1) Å, Q_T_ = 0.569 (1) Å, φ = -128.0 (1)° for cyclo­hexane ring. In the piperidine ring , atoms N1 and C3 deviate at 0.639 (1) and -0.705 (1) Å, respectively, from the least-squares plane formed by the remaining four atoms, whereas in cyclo­hexane ring, atoms C19 and C3 deviate at 0.562 (1) and -0.721 (1) Å, respectively, from the least-squares plane formed by the remaining four atoms.

### S3. Supra­molecular features

The crystal packing is stabilized by van der Waals forces only, since the amino group is not involved in any hydrogen-bonding inter­actions.

### S4. Synthesis and crystallization

To a solution of the 2, 4-di­phenyl-3-aza­bicyclo [3.3.1] nonan-9-one (500 mg, 1.72 mmol) in THF (5 mL), LiOH (212 mg, 3.516mmol) and di­ethyl­cyano­methyl phospho­nate (364g, 1.4063mmol) was added. The reaction mixture was stirred at for 3 h. After completion of the reaction (monitored by TLC), the reaction mixture was diluted with ethyl acetate (45 mL). The organic layer was washed with water (10 mL X 3) and dried over Na_2_SO_4_. The filtrate was concentrated and the crude product mass was purified by column-chromatography over silica-gel (100–200 mesh) using petroleum ether and di­ethyl ether (5-10%) as eluent to give a colorless solid. This solid was recrystallized in ethyl acetate to yield a colourless crystals of the title compound.

### S5. Refinement

Atom H1N was located from a difference Fourier map and refined with a bond length restraint of 0.90 (2) Å. The remaining H atoms were positioned geometrically and were treated as riding on their parent C atoms, with C—H distances of 0.93-0.98 Å, and U_iso_(H) = 1.5U_eq_ (C) for methyl H and U_iso_(H) = 1.2U_eq_(C) for all other H atoms.

### Crystal data

**Table:**

C_22_H_22_N_2_	*Z* = 2
*M**_r_* = 314.41	*F*(000) = 336
Triclinic, *P*1	*D*_x_ = 1.176 Mg m^−^^3^
*a* = 7.9672 (5) Å	Mo *K*α radiation, λ = 0.71073 Å
*b* = 8.3129 (5) Å	Cell parameters from 9878 reflections
*c* = 13.6069 (8) Å	θ = 2.3–27.2°
α = 89.607 (4)°	µ = 0.07 mm^−^^1^
β = 81.886 (4)°	*T* = 296 K
γ = 84.469 (4)°	Block, colourless
*V* = 888.00 (9) Å^3^	0.23 × 0.21 × 0.19 mm

### Data collection

**Table:**

Bruker SMART APEX CCD area-detector diffractometer	*R*_int_ = 0.034
Radiation source: fine-focus sealed tube	θ_max_ = 27.2°, θ_min_ = 1.5°
ω scans	*h* = −10→10
14326 measured reflections	*k* = −9→10
3814 independent reflections	*l* = −17→17
2375 reflections with *I* > 2σ(*I*)	

### Refinement

**Table:**

Refinement on *F*^2^	0 restraints
Least-squares matrix: full	Hydrogen site location: mixed
*R*[*F*^2^ > 2σ(*F*^2^)] = 0.047	H atoms treated by a mixture of independent and constrained refinement
*wR*(*F*^2^) = 0.131	*w* = 1/[σ^2^(*F*_o_^2^) + (0.0623*P*)^2^ + 0.0228*P*] where *P* = (*F*_o_^2^ + 2*F*_c_^2^)/3
*S* = 1.05	(Δ/σ)_max_ < 0.001
3814 reflections	Δρ_max_ = 0.13 e Å^−^^3^
221 parameters	Δρ_min_ = −0.16 e Å^−^^3^

### Special details

**Table:**

Geometry. All esds (except the esd in the dihedral angle between two l.s. planes) are estimated using the full covariance matrix. The cell esds are taken into account individually in the estimation of esds in distances, angles and torsion angles; correlations between esds in cell parameters are only used when they are defined by crystal symmetry. An approximate (isotropic) treatment of cell esds is used for estimating esds involving l.s. planes.

### Fractional atomic coordinates and isotropic or equivalent isotropic displacement parameters (Å^2^)

**Table:**

	*x*	*y*	*z*	*U*_iso_*/*U*_eq_	
N1	0.30954 (14)	0.47358 (15)	0.20403 (9)	0.0491 (3)	
H1N	0.3354 (19)	0.5473 (19)	0.1565 (12)	0.069 (5)*	
N2	0.6722 (2)	0.17287 (19)	0.51383 (11)	0.0845 (5)	
C1	0.21406 (17)	0.55407 (17)	0.29305 (10)	0.0494 (4)	
H1A	0.2864	0.6291	0.3178	0.059*	
C2	0.17259 (18)	0.42725 (18)	0.37503 (11)	0.0543 (4)	
H2	0.1209	0.4846	0.4362	0.065*	
C3	0.33678 (17)	0.33590 (17)	0.39353 (10)	0.0518 (4)	
C4	0.42543 (17)	0.24949 (17)	0.30202 (10)	0.0503 (4)	
H4	0.5326	0.1933	0.3171	0.060*	
C5	0.46745 (16)	0.37958 (17)	0.22222 (10)	0.0465 (3)	
H5	0.5395	0.4532	0.2485	0.056*	
C6	0.05227 (17)	0.65065 (17)	0.27059 (11)	0.0511 (4)	
C7	−0.0303 (2)	0.6131 (2)	0.19207 (13)	0.0661 (5)	
H7	0.0167	0.5294	0.1487	0.079*	
C8	−0.1827 (2)	0.6993 (2)	0.17744 (15)	0.0808 (5)	
H8	−0.2375	0.6731	0.1246	0.097*	
C9	−0.2528 (2)	0.8236 (2)	0.24123 (16)	0.0825 (6)	
H9	−0.3550	0.8814	0.2314	0.099*	
C10	−0.1720 (2)	0.8623 (2)	0.31933 (15)	0.0730 (5)	
H10	−0.2195	0.9459	0.3626	0.088*	
C11	−0.01988 (19)	0.77658 (18)	0.33353 (12)	0.0607 (4)	
H11	0.0348	0.8039	0.3862	0.073*	
C12	0.56488 (17)	0.30391 (16)	0.12778 (10)	0.0475 (3)	
C13	0.48803 (19)	0.2693 (2)	0.04674 (11)	0.0614 (4)	
H13	0.3721	0.2981	0.0478	0.074*	
C14	0.5812 (2)	0.1923 (2)	−0.03620 (12)	0.0710 (5)	
H14	0.5270	0.1690	−0.0899	0.085*	
C15	0.7520 (2)	0.1501 (2)	−0.03997 (13)	0.0717 (5)	
H15	0.8139	0.0983	−0.0959	0.086*	
C16	0.8314 (2)	0.1848 (2)	0.03951 (14)	0.0772 (5)	
H16	0.9478	0.1571	0.0375	0.093*	
C17	0.73838 (18)	0.2608 (2)	0.12246 (12)	0.0660 (5)	
H17	0.7933	0.2837	0.1759	0.079*	
C18	0.05238 (18)	0.3031 (2)	0.35037 (12)	0.0643 (4)	
H18A	0.0231	0.2383	0.4087	0.077*	
H18B	−0.0520	0.3606	0.3343	0.077*	
C19	0.12981 (19)	0.19133 (19)	0.26393 (12)	0.0634 (4)	
H19A	0.1298	0.2507	0.2023	0.076*	
H19B	0.0597	0.1028	0.2612	0.076*	
C20	0.31190 (19)	0.12269 (17)	0.27312 (12)	0.0588 (4)	
H20A	0.3635	0.0737	0.2102	0.071*	
H20B	0.3080	0.0382	0.3227	0.071*	
C21	0.39261 (19)	0.33330 (18)	0.48167 (11)	0.0575 (4)	
H21	0.3278	0.3924	0.5339	0.069*	
C22	0.5476 (2)	0.2435 (2)	0.49907 (11)	0.0613 (4)	

### Atomic displacement parameters (Å^2^)

**Table:**

	*U*^11^	*U*^22^	*U*^33^	*U*^12^	*U*^13^	*U*^23^
N1	0.0458 (7)	0.0516 (7)	0.0477 (7)	0.0043 (5)	−0.0046 (5)	0.0050 (6)
N2	0.0855 (11)	0.0958 (12)	0.0753 (10)	0.0097 (9)	−0.0348 (8)	0.0004 (9)
C1	0.0453 (8)	0.0515 (8)	0.0503 (9)	0.0025 (6)	−0.0075 (6)	−0.0030 (7)
C2	0.0505 (8)	0.0635 (10)	0.0454 (8)	0.0066 (7)	−0.0029 (6)	−0.0003 (7)
C3	0.0515 (8)	0.0557 (9)	0.0477 (9)	−0.0010 (7)	−0.0084 (7)	0.0047 (7)
C4	0.0473 (8)	0.0552 (9)	0.0479 (8)	0.0069 (6)	−0.0127 (6)	0.0020 (7)
C5	0.0407 (7)	0.0513 (8)	0.0474 (8)	−0.0005 (6)	−0.0084 (6)	−0.0022 (7)
C6	0.0474 (8)	0.0488 (8)	0.0552 (9)	0.0015 (6)	−0.0043 (7)	0.0033 (7)
C7	0.0596 (10)	0.0666 (10)	0.0712 (11)	0.0116 (8)	−0.0180 (8)	−0.0069 (9)
C8	0.0714 (11)	0.0835 (13)	0.0895 (14)	0.0119 (9)	−0.0310 (10)	−0.0014 (11)
C9	0.0612 (11)	0.0734 (12)	0.1101 (16)	0.0195 (9)	−0.0200 (10)	0.0072 (11)
C10	0.0662 (11)	0.0580 (10)	0.0882 (13)	0.0135 (8)	−0.0014 (9)	−0.0027 (9)
C11	0.0609 (9)	0.0528 (9)	0.0658 (10)	0.0046 (7)	−0.0068 (8)	−0.0015 (8)
C12	0.0451 (8)	0.0486 (8)	0.0479 (8)	−0.0007 (6)	−0.0067 (6)	0.0008 (7)
C13	0.0540 (9)	0.0757 (11)	0.0533 (10)	0.0053 (8)	−0.0112 (7)	−0.0042 (8)
C14	0.0782 (12)	0.0826 (12)	0.0512 (10)	0.0030 (9)	−0.0127 (8)	−0.0090 (9)
C15	0.0770 (12)	0.0732 (11)	0.0576 (10)	0.0086 (9)	0.0052 (9)	−0.0101 (9)
C16	0.0508 (9)	0.0956 (14)	0.0791 (13)	0.0118 (9)	−0.0002 (9)	−0.0140 (11)
C17	0.0487 (9)	0.0839 (12)	0.0641 (11)	0.0048 (8)	−0.0100 (7)	−0.0135 (9)
C18	0.0487 (9)	0.0721 (11)	0.0706 (11)	−0.0041 (7)	−0.0051 (7)	0.0172 (9)
C19	0.0581 (9)	0.0599 (10)	0.0754 (11)	−0.0145 (7)	−0.0147 (8)	0.0076 (9)
C20	0.0652 (10)	0.0510 (9)	0.0601 (10)	−0.0017 (7)	−0.0112 (7)	0.0066 (8)
C21	0.0621 (9)	0.0621 (10)	0.0472 (9)	0.0014 (7)	−0.0094 (7)	0.0002 (7)
C22	0.0708 (11)	0.0681 (10)	0.0478 (9)	−0.0033 (8)	−0.0204 (8)	0.0011 (8)

### Geometric parameters (Å, º)

**Table:**

N1—C1	1.4651 (17)	C10—C11	1.383 (2)
N1—C5	1.4665 (16)	C10—H10	0.9300
N1—H1N	0.904 (16)	C11—H11	0.9300
N2—C22	1.1461 (18)	C12—C13	1.379 (2)
C1—C6	1.5191 (18)	C12—C17	1.3860 (18)
C1—C2	1.553 (2)	C13—C14	1.385 (2)
C1—H1A	0.9800	C13—H13	0.9300
C2—C3	1.5000 (18)	C14—C15	1.365 (2)
C2—C18	1.543 (2)	C14—H14	0.9300
C2—H2	0.9800	C15—C16	1.373 (2)
C3—C21	1.3361 (19)	C15—H15	0.9300
C3—C4	1.4939 (19)	C16—C17	1.381 (2)
C4—C20	1.541 (2)	C16—H16	0.9300
C4—C5	1.5529 (19)	C17—H17	0.9300
C4—H4	0.9800	C18—C19	1.526 (2)
C5—C12	1.5116 (18)	C18—H18A	0.9700
C5—H5	0.9800	C18—H18B	0.9700
C6—C7	1.384 (2)	C19—C20	1.5286 (19)
C6—C11	1.385 (2)	C19—H19A	0.9700
C7—C8	1.388 (2)	C19—H19B	0.9700
C7—H7	0.9300	C20—H20A	0.9700
C8—C9	1.378 (2)	C20—H20B	0.9700
C8—H8	0.9300	C21—C22	1.428 (2)
C9—C10	1.373 (3)	C21—H21	0.9300
C9—H9	0.9300		
			
C1—N1—C5	113.55 (11)	C11—C10—H10	120.1
C1—N1—H1N	110.1 (10)	C10—C11—C6	121.05 (16)
C5—N1—H1N	108.7 (9)	C10—C11—H11	119.5
N1—C1—C6	111.71 (11)	C6—C11—H11	119.5
N1—C1—C2	109.87 (11)	C13—C12—C17	117.63 (14)
C6—C1—C2	110.76 (11)	C13—C12—C5	123.02 (12)
N1—C1—H1A	108.1	C17—C12—C5	119.30 (12)
C6—C1—H1A	108.1	C12—C13—C14	120.89 (14)
C2—C1—H1A	108.1	C12—C13—H13	119.6
C3—C2—C18	107.86 (12)	C14—C13—H13	119.6
C3—C2—C1	108.18 (11)	C15—C14—C13	120.67 (15)
C18—C2—C1	115.18 (12)	C15—C14—H14	119.7
C3—C2—H2	108.5	C13—C14—H14	119.7
C18—C2—H2	108.5	C14—C15—C16	119.43 (15)
C1—C2—H2	108.5	C14—C15—H15	120.3
C21—C3—C4	125.54 (13)	C16—C15—H15	120.3
C21—C3—C2	123.15 (14)	C15—C16—C17	119.93 (15)
C4—C3—C2	111.31 (11)	C15—C16—H16	120.0
C3—C4—C20	108.48 (12)	C17—C16—H16	120.0
C3—C4—C5	107.33 (11)	C16—C17—C12	121.45 (15)
C20—C4—C5	115.21 (11)	C16—C17—H17	119.3
C3—C4—H4	108.6	C12—C17—H17	119.3
C20—C4—H4	108.6	C19—C18—C2	113.21 (12)
C5—C4—H4	108.6	C19—C18—H18A	108.9
N1—C5—C12	111.71 (11)	C2—C18—H18A	108.9
N1—C5—C4	109.50 (10)	C19—C18—H18B	108.9
C12—C5—C4	111.25 (11)	C2—C18—H18B	108.9
N1—C5—H5	108.1	H18A—C18—H18B	107.7
C12—C5—H5	108.1	C18—C19—C20	112.39 (13)
C4—C5—H5	108.1	C18—C19—H19A	109.1
C7—C6—C11	118.53 (14)	C20—C19—H19A	109.1
C7—C6—C1	122.50 (13)	C18—C19—H19B	109.1
C11—C6—C1	118.91 (13)	C20—C19—H19B	109.1
C6—C7—C8	120.54 (16)	H19A—C19—H19B	107.9
C6—C7—H7	119.7	C19—C20—C4	113.88 (12)
C8—C7—H7	119.7	C19—C20—H20A	108.8
C9—C8—C7	120.01 (17)	C4—C20—H20A	108.8
C9—C8—H8	120.0	C19—C20—H20B	108.8
C7—C8—H8	120.0	C4—C20—H20B	108.8
C10—C9—C8	120.03 (16)	H20A—C20—H20B	107.7
C10—C9—H9	120.0	C3—C21—C22	122.75 (14)
C8—C9—H9	120.0	C3—C21—H21	118.6
C9—C10—C11	119.84 (17)	C22—C21—H21	118.6
C9—C10—H10	120.1	N2—C22—C21	179.18 (18)
			
C5—N1—C1—C6	179.66 (11)	C6—C7—C8—C9	0.1 (3)
C5—N1—C1—C2	−57.00 (15)	C7—C8—C9—C10	0.0 (3)
N1—C1—C2—C3	55.94 (15)	C8—C9—C10—C11	0.3 (3)
C6—C1—C2—C3	179.83 (11)	C9—C10—C11—C6	−0.6 (3)
N1—C1—C2—C18	−64.80 (15)	C7—C6—C11—C10	0.7 (2)
C6—C1—C2—C18	59.09 (16)	C1—C6—C11—C10	−176.43 (14)
C18—C2—C3—C21	−115.00 (16)	N1—C5—C12—C13	−25.14 (19)
C1—C2—C3—C21	119.81 (16)	C4—C5—C12—C13	97.57 (16)
C18—C2—C3—C4	64.47 (15)	N1—C5—C12—C17	157.44 (13)
C1—C2—C3—C4	−60.72 (15)	C4—C5—C12—C17	−79.85 (16)
C21—C3—C4—C20	116.21 (16)	C17—C12—C13—C14	0.8 (2)
C2—C3—C4—C20	−63.25 (14)	C5—C12—C13—C14	−176.61 (14)
C21—C3—C4—C5	−118.71 (15)	C12—C13—C14—C15	−0.6 (3)
C2—C3—C4—C5	61.83 (14)	C13—C14—C15—C16	0.0 (3)
C1—N1—C5—C12	−177.87 (11)	C14—C15—C16—C17	0.4 (3)
C1—N1—C5—C4	58.42 (15)	C15—C16—C17—C12	−0.1 (3)
C3—C4—C5—N1	−58.56 (14)	C13—C12—C17—C16	−0.5 (2)
C20—C4—C5—N1	62.36 (15)	C5—C12—C17—C16	177.05 (15)
C3—C4—C5—C12	177.47 (10)	C3—C2—C18—C19	−55.28 (16)
C20—C4—C5—C12	−61.61 (14)	C1—C2—C18—C19	65.64 (16)
N1—C1—C6—C7	25.8 (2)	C2—C18—C19—C20	46.74 (17)
C2—C1—C6—C7	−97.05 (17)	C18—C19—C20—C4	−45.54 (17)
N1—C1—C6—C11	−157.20 (13)	C3—C4—C20—C19	52.91 (15)
C2—C1—C6—C11	79.97 (16)	C5—C4—C20—C19	−67.39 (16)
C11—C6—C7—C8	−0.5 (2)	C4—C3—C21—C22	−0.6 (2)
C1—C6—C7—C8	176.55 (14)	C2—C3—C21—C22	178.80 (14)
