# Supplementary figures and images for: Crystal structure of 2-(2,4-diphenyl-3-aza­bicyclo­[3.3.1]nonan-9-yl­idene)aceto­nitrile
Source: Acta Crystallogr E Crystallogr Commun. 2015 Sep 26;71(Pt 10):o792–3. doi: 10.1107/S2056989015017740 (PMC4647352; doi:10.1107/S2056989015017740)

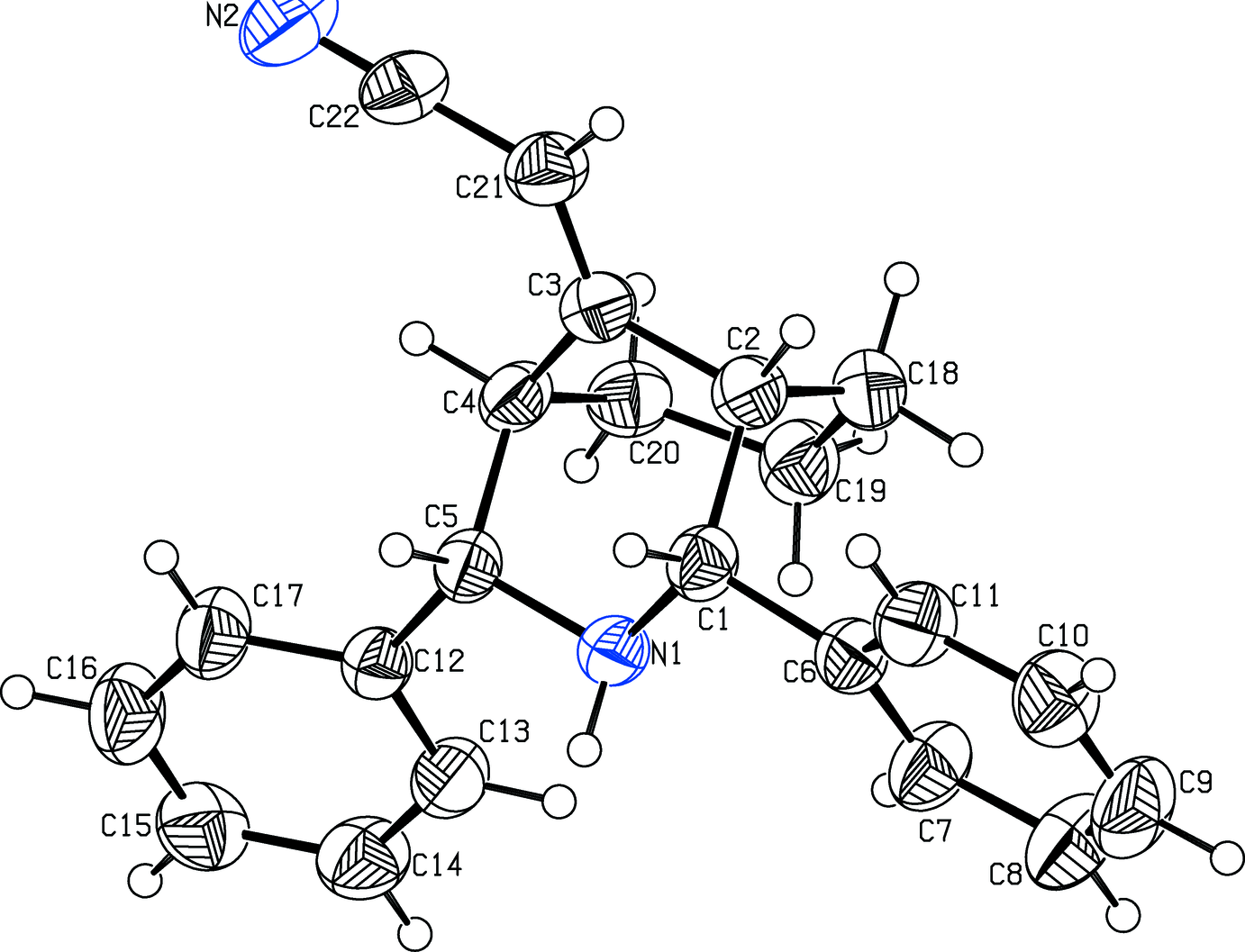

Supplement: Supplementary file 4 [file e-71-0o792-fig1.tif]
